# Interleukins 4 and 13 in Asthma: Key Pathophysiologic Cytokines and Druggable Molecular Targets

**DOI:** 10.3389/fphar.2022.851940

**Published:** 2022-03-08

**Authors:** Corrado Pelaia, Enrico Heffler, Claudia Crimi, Angelantonio Maglio, Alessandro Vatrella, Girolamo Pelaia, Giorgio Walter Canonica

**Affiliations:** ^1^ Department of Health Sciences, University “Magna Græcia” of Catanzaro, Catanzaro, Italy; ^2^ Personalized Medicine, Asthma and Allergy, Humanitas Clinical and Research Center IRCCS, Rozzano, Italy; ^3^ Department of Biomedical Sciences, Humanitas University, Pieve Emanuele, Italy; ^4^ Department of Clinical and Experimental Medicine, University of Catania, Catania, Italy; ^5^ Department of Medicine, Surgery, and Dentistry, University of Salerno, Salerno, Italy

**Keywords:** severe asthma, IL-4, IL-13, dupilumab, IL-4 receptor

## Abstract

Interleukins (IL)-4 and -13 play a pivotal role in the pathobiology of type-2 asthma. Indeed, IL-4 is crucially involved in Th2 cell differentiation, immunoglobulin (Ig) class switching and eosinophil trafficking. IL-13 cooperates with IL-4 in promoting IgE synthesis, and also induces nitric oxide (NO) production, goblet cell metaplasia and fibroblast proliferation, as well as elicits contractile responses and hyperplasia of airway smooth muscle cells.

IL-4 and IL-13 share common signaling pathways, activated by the binding of both cytokines to receptor complexes including the α-subunit of the IL-4 receptor (IL-4Rα). Therefore, the subsequent receptor dimerization is responsible for the pathophysiologic effects of IL-4 and IL-13. By selectively blocking IL-4Rα, the fully human IgG4 monoclonal antibody dupilumab behaves as a dual receptor antagonist of both IL-4 and IL-13. Through this mechanism of action, dupilumab exerts effective therapeutic actions in type-2 inflammation, thus decreasing asthma exacerbations, FeNO (fractional exhaled NO) levels, and the intake of oral corticosteroids (OCS). In addition to being approved for the add-on biological therapy of severe asthma, dupilumab has also been licensed for the treatment of nasal polyposis and atopic dermatitis.

## Introduction

**FIGURE 1:**
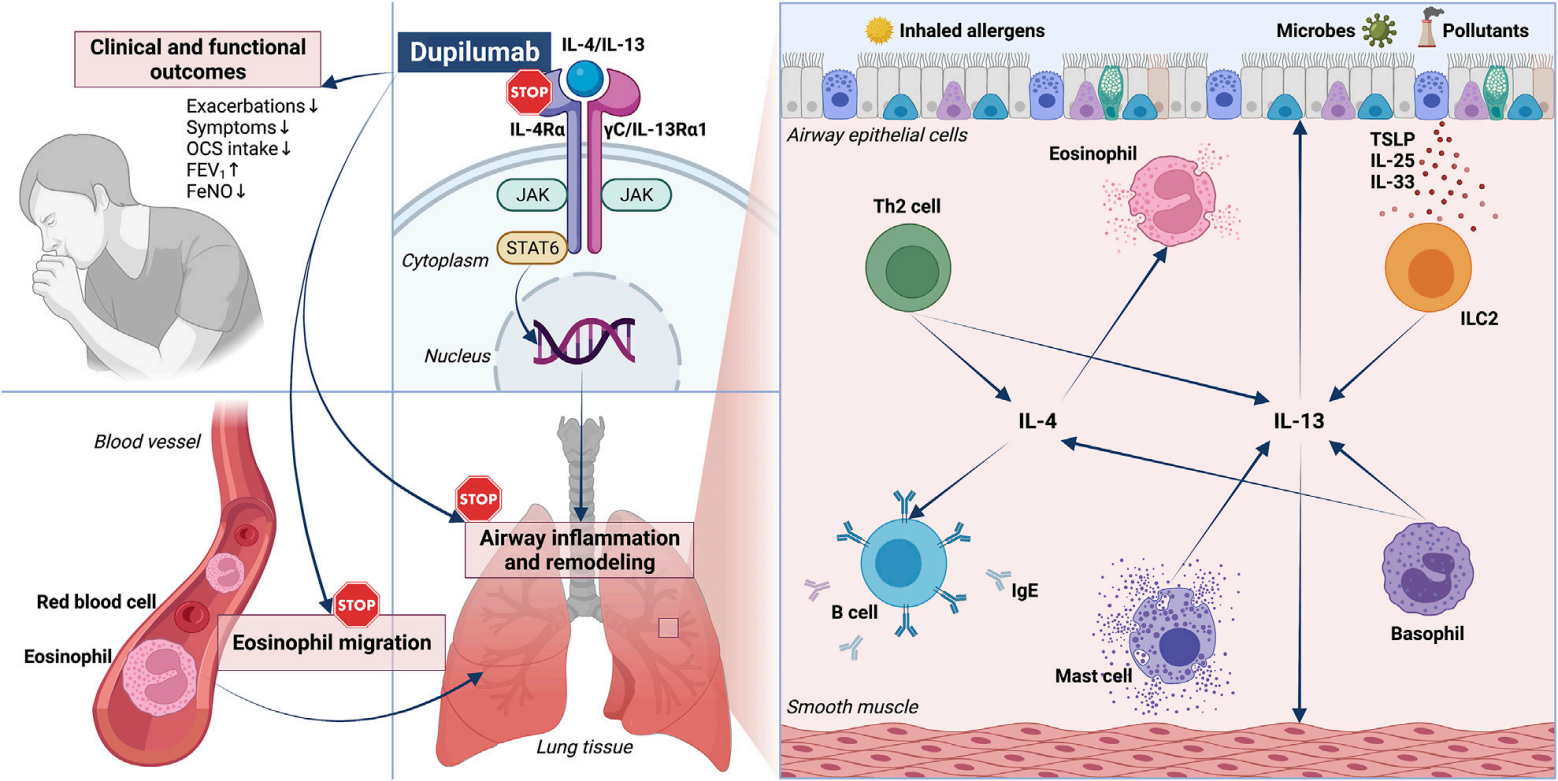
Pathophysiology and biological therapy of type 2 asthma: respective roles of IL-4/IL-13 and dual receptor blockade by dupilumab. RIGHT PANEL—A key role in the pathobiology of type 2 asthma is played by both IL-4 and IL-13, released by Th2 lymphocytes, ILC2, basophils, and mast cells. IL-4 and IL-13 act on many cellular targets, including immune inflammatory cells such as B lymphocytes and eosinophils, as well as airway structural cells (epithelial cells and smooth muscle cells). LEFT PANEL–By binding to IL-4Rα, dupilumab operates a receptor blockade which suppresses the biological effects of IL-4 and IL-13, and is thus responsible for several therapeutic effects including clinical and functional improvements, as well as inhibition of eosinophil trafficking. IL-4Rα: α subunit of IL-4 receptor; IL-13Rα1: α1 subunit of IL-13 receptor; JAK: Janus kinase; STAT: signal transducer and activator of transcription; TSLP: thymic stromal lymphopoietin; ILC2: group 2 innate lymphoid cells.

Asthma is a widespread respiratory disorder, usually characterized by variable airflow limitation associated with airway inflammation and remodeling (Holgate et al., 2015; Khalaf et al., 2019; Stern et al., 2020). This heterogeneous disease includes several phenotypes, originating from complex interactions between genetic, and environmental factors (Kuruvilla et al., 2019; Thomsen, 2015). The different asthmatic phenotypes are mainly shaped by distinct inflammatory profiles, underpinned by intricate networks of cellular and molecular pathomechanisms known as endotypes, which can also be responsible for the development of severe clinical features (Komlosi et al., 2022; Wenzel, 2021). In particular, the multiple variants of asthma may consist of either eosinophilic, neutrophilic, mixed, or paucigranulocytic patterns (Papi et al., 2018; Suraya et al., 2021; Carr et al., 2018; Tliba and Panettieri, 2019). Eosinophilic airway infiltration is the dominant effector inflammatory trait of type-2 (T2) allergic or non-allergic asthma, driven by interacting innate and adaptive immune responses orchestrated by group 2 innate lymphoid cells (ILC2) and T helper 2 (Th2) lymphocytes, producing the interleukins 4 (IL-4), 13 (IL-13), and 5 (IL-5) (Nelson et al., 2020; Rodriguez-Rodriguez et al., 2021; Hammad and Lambrecht, 2021). In type-2 asthma, these cytokines exert key functions with regard to inception, persistence and amplification of bronchial inflammation and remodeling. Indeed, IL-4 promotes both Th2 cell differentiation and biosynthesis of immunoglobulins E (IgE), whereas IL-13 is mostly responsible for airway hyperresponsiveness, mucus overproduction, and bronchial structural changes (Figure 1) (Matucci et al., 2021). Moreover, IL-5 is the principal inducer of maturation, survival, proliferation, and activation of eosinophils (Pelaia et al., 2019). Within such a pathogenic context, a crucial role is played by tissue damage occurring at level of the bronchial epithelium, which releases copious amounts of alarmins when injured by one or more of many triggers including allergens, cigarette smoke, airborne pollutants, and infectious agents (Calvén et al., 2020). Alarmins comprehend the innate cytokines thymic stromal lymphopoietin (TSLP), interleukin-25 (IL-25) and interleukin-33 (IL-33), which act as upstream stimulators of both innate, and adaptive immune cascades implicated in type-2 asthma (Hong et al., 2020). In fact, alarmins directly activate ILC2, and also prime conventional dendritic cells to elicit the maturation and clonal expansion of Th2 lymphocytes (Hammad and Lambrecht, 2021; Komlosi et al., 2022). As a consequence, alarmins induce the release of type-2 cytokines such as IL-13, which in turn enhances airway epithelial cell secretion of IL-33, thereby nurturing a feed-forward pathogenic circuit that significantly amplifies type-2 inflammation in asthmatic patients (Christianson et al., 2015).

The above arguments explain why IgE, IL-4/IL-13 receptors, IL-5 and its receptor, as well as alarmins and their receptors represent suitable molecular targets for current and prospective biological therapies of severe asthma (Pelaia et al., 2020; Pelaia et al., 2022; Busse et al., 2021; Porsbjerg et al., 2020; Albrecht, 2021). In particular, the fully human monoclonal antibody dupilumab behaves as a dual IL-4/IL-13 receptor antagonist, which according to both randomized trials and real-life studies has been shown to effectively inhibit type-2 inflammation, thus significantly improving several clinical and functional parameters related to asthma (Figure 1), nasal polyposis, and atopic dermatitis (Pelaia et al., 2017; Ricciardolo et al., 2021).

Therefore, the present narrative review aims to provide an updated coverage of the following two topics: 1) pathobiologic roles of IL-4 and IL-13 in type-2 asthma; 2) therapeutic effects of dupilumab in severe asthma.

## Pathobiologic Roles of IL-4 and IL-13 in Type 2 Asthma

The main cellular sources of IL-4 and IL-13 include Th2 lymphocytes, T follicular helper (Tfh) cells and ILC2, and a significant contribution to the production of these two cytokines is also given by eosinophils, basophils, mast cells, natural killer cells, and CD8^+^ T lymphocytes (Steinke and Borish, 2001; Vijayanand et al., 2012; Corren, 2013; Maggi et al., 2017; Gowthaman et al., 2019; Komlosi et al., 2022). In regard to the pathobiology of type-2 allergic asthma, IL-4 and IL-13 play relevant roles in airway inflammation and remodeling (Komlosi et al., 2022). Initially released from basophils, IL-4 is essential for differentiation of Th2 cells derived from naïve CD4^+^ T lymphocytes (Kaiko et al., 2008; Gandhi et al., 2016). This pathogenic task of IL-4 is further favored by its ability to suppress the immunomodulatory and tolerogenic functions of regulatory T (Treg) cells (Tu et al., 2017), which do not allow Th2 cell maturation and clonal expansion in non-allergic subjects (Palomares et al., 2017). Together with IL-13, IL-4 provides the so-called “first signal” that induces IgE production by B cells (Geha et al., 2003; Froidure et al., 2016). IgE class switching also requires a “second signal” for maturation of B lymphocytes, expressed by the B-T cell cognate interaction consisting of an immunological synapse engaging both T cell receptor (TCR) and CD40/CD40 ligand (CD40L) coupling (Novosad and Krčmová, 2020). Although Th2 and Tfh cells are probably the most relevant cellular sources of IL-4 and IL-13, these cytokines are also synthesized by ILC2, which thus contribute to stimulate IgE production (Maggi et al., 2017). Upon binding of allergens to adjacent IgE molecules anchored to their high affinity receptors (FcεRI) located on the surface of mast cells and basophils, these cells secrete large amounts of IL-4 and IL-13 (Stone et al., 2010; Komlosi et al., 2022), which in turn further amplify the intensity of type-2 airway inflammation.

In addition to T and B lymphocytes, IL-4 and IL-13 act on other cellular targets including both immune/inflammatory and airway structural cells involved in asthma pathophysiology (Figure 1). In particular, IL-13 up-regulates FcεRI expression on mast cells, and raises their proliferation (Marone et al., 2019). The most abundant immune cells in the lung are macrophages, which express IL-4/IL-13 receptors whose stimulation drives the polarization towards the M2 macrophage subtype, actively involved in the pathogenesis of severe allergic asthma (Abdelaziz et al., 2020; Becerra-Díaz et al., 2021). IL-4 promotes the attachment of eosinophils to blood vessel walls by enhancing the expression of vascular cell adhesion molecule-1 (VCAM-1), and IL-13 contributes to eosinophil recruitment into the airways by increasing eotaxin synthesis at level of the bronchial epithelium (Rosenberg et al., 2007; Nagata et al., 2020). This tissue is disrupted by IL-13, which down-regulates the expression of claudin-18.1, an important protein component of the intercellular tight junctions that maintain the physical integrity of the airway epithelial barrier (Sweerus et al., 2017). Moreover, IL-4 and IL-13 enhance the expression of histone deacetylases 1 and 9 (HDAC 1 and 9), whose activity is inversely correlated to the integrity of the airway epithelial cell layer (Steelant et al., 2019). Therefore, both IL-4 and IL-13 significantly participate in the induction of bronchial epithelial dysfunction, which is a hallmark of asthma. IL-13 exerts further effects on airway epithelial cells, where this cytokine elicits goblet cell metaplasia and the production of the glycoprotein mucin 5AC (MUC5AC), which is associated with a more viscous type of mucus (Dickinson et al., 2016). Additionally, in bronchial epithelial cells IL-13 stimulates the expression of the inducible isoform of nitric oxide synthase (iNOS), thereby increasing the airway levels of nitric oxide (Ricciardolo and Silkoff, 2017), a reliable biomarker of type 2 asthma. With regard to airway mesenchymal tissues, IL-13 causes contraction, and proliferation of airway smooth muscle cells (Corren, 2013; Busse et al., 2021). Moreover, IL-13 is a pivotal inducer of bronchial sub-epithelial fibrosis due to fibroblast proliferation and collagen production, and these effects are at least in part mediated by IL-13-dependent activation of transforming growth factor-β1 (TGF-β1) (Lee et al., 2001; Firszt et al., 2014). Such structural changes express the powerful action of IL-13 as a central mediator of airway remodeling in asthma.

Further evidence referring to the very important roles played by IL-4 and IL-13 in asthma pathobiology ensues from the copious quantities of these cytokines which can be found in bronchial mucosa, induced sputum, bronchoalveolar lavage (BAL) fluid, and peripheral blood from asthmatic patients (Saha et al., 2008; Maes et al., 2012; Corren, 2013). Moreover, exposure of asthmatic subjects to segmental allergen challenges significantly up-regulated airway levels of IL-4/IL-13 mRNAs (Prieto et al., 2000; Saha et al., 2008; Maes et al., 2012; Corren, 2013). Genetic investigations have also detected relevant linkages of IL-13/IL-13 receptor gene polymorphisms with airway hyperresponsiveness and asthma prevalence (Howard et al., 2002). It is thus possible that the genetic control of IL-13 contributes to shape the individual susceptibility to asthma, which is associated with some polymorphisms identified in the RAD50-IL-13 region of chromosome 5q31.1 (Li et al., 2010).

Even if the functional roles of IL-4 and IL-13 are quite overlapping, it is however plausible that these two sister cytokines exert distinct pathobiological actions in asthma. In fact, IL-4 is the key inducer of CD4^+^ Th cell commitment towards a Th2 immunophenotype, whilst IL-13 primarily promotes the development of bronchial inflammation and remodeling, thus enhancing airway hyperresponsiveness (Coyle et al., 1995; Grünig et al., 1998; Wills-Karp et al., 1998). Indeed, in comparison to IL-4, higher levels of IL-13 can be found in the airways of asthmatic patients, as well as in murine lungs sensitized to allergens (Kotsimbos et al., 1996; Munitz et al., 2008).

IL-4 and IL-13 exert their biologic functions by interacting with specific receptor structures expressed on several target cells (Nelms et al., 1999; Ul-Haq et al., 2016; Chatila, 2004). In particular, only IL-4 binds to type I receptor, a heterodimeric complex in which the IL-4 receptor α-subunit (IL-4Rα) is coupled with the γC chain (Figure 1) (Nelms et al., 1999). Both IL-4 and IL-13 can interact with type II receptor, a heterodimer consisting of the IL-4Rα component paired with the IL-13 receptor α1-subunit (IL-13Rα1) (McCormick and Heller, 2015).

Binding of IL-4 to type I receptor triggers the activation of Janus kinases 1 (JAK1) and 3 (JAK3), which are linked to the receptor cytoplasmic domains (Wills-Karp and Finkelman, 2008). JAK1 and JAK3 then catalyze the phosphorylation of the tyrosine residues Y500, Y575, Y603, and Y633 localized in the intracellular region of the IL-4Rα subunit, where these phosphorylated amino acids act as a signaling module which recruits key transducing proteins such as insulin receptor substrate-2 (IRS-2) and signal transducer and activator of transcription-6 (STAT-6) (Kelly-Welch et al., 2003; Wills-Karp and Finkelman, 2008; Oh et al., 2010; Gour and Wills-Karp, 2015; Harb and Chatila, 2020). IRS-2 proteins bind to the p85 subunit of phosphoinositide-3 kinase (PI3K) and to the adaptor protein growth factor receptor-bound protein 2 (Grb2), which are associated with the PI3K/AKT signaling pathway involved in Th2 cell proliferation (Gour and Wills-Karp, 2015; Pelaia et al., 2017; Harb and Chatila, 2020). Furthermore, as a result of JAK1/JAK3-mediated tyrosine phosphorylation, STAT-6 undergoes activation, dimerization and nuclear translocation, thus enabling the transcription factor GATA-3 to up-regulate the expression of the genes encoding IL-4, IL-5, and IL-13 (Zeng, 2013; Tindemans et al., 2014; Pelaia et al., 2017; Massey and Suphioglu, 2021).

The type II heterodimeric receptor, composed of IL-4Rα/IL-13Rα1 subunits and activated by IL-4 and IL-13, is functionally coupled with JAK1/2, tyrosine kinase 2 (Tyk2), and STAT-6, but is not associated with JAK3 and IRS-2 (Andrews et al., 2001; Chiba et al., 2012). IL-13 is also the endogenous ligand of the IL-13 receptor α2-chain (IL-13Rα2), which is dissociated from any other receptor protein or signaling network, thereby being responsible for a negative feedback mechanism which inhibits IL-13 functions (Zheng et al., 2008).

## Therapeutic Effects of Dupilumab in Severe Asthma

The fully human IgG4 monoclonal antibody dupilumab was realized by Sanofi (Gentilly, France) and Regeneron Pharmaceuticals (Tarrytown, NY, United States) (Shirley, 2017; Santini et al., 2017). Dupilumab specifically binds to IL-4Rα, thereby preventing the interaction between IL-4, and the type I receptor complex (Figure 1) (Vatrella et al., 2014). It is also likely that such an IL-4Rα blockade can make it possible for dupilumab to inhibit the recruitment of this subunit, triggered by the interaction of IL-13 with IL-13Rα1, and responsible for the assembly of type II receptor (Harb and Chatila, 2020). Therefore, dupilumab behaves as a dual receptor antagonist of IL-4 and IL-13.

Many phase 1 trials were conducted in healthy subjects in order to evaluate the safety and tolerability profile of dupilumab, as well as its pharmacokinetics and immunogenicity (Santini et al., 2017). After subcutaneous administration, dupilumab exhibited a non-linear pharmacokinetics, featured by higher than dose-proportional increments of systemic exposure (Kovalenko et al., 2016; Shirley, 2017). Subsequently to a 600 mg subcutaneous injection, dupilumab displayed an approximate bioavailability of 64%, a total distribution volume of about 4.8 L, and a mean peak concentration at 1 week of 70.1 μg/ml (Vatrella et al., 2014).

The first phase 2a double-blind, randomized, and placebo-controlled trial aimed to assess the therapeutic effects of dupilumab in asthma was carried out in 104 patients (age range: 18–65 years) with moderate-to-severe asthma, characterized by relatively high levels of eosinophils in both blood (≥300 cells/μl) and sputum (≥3%) (Wenzel et al., 2013). The inhaled treatment consisted of medium/high doses of inhaled corticosteroids (ICS) and long-acting β_2_-adrenergic agonists (LABA), which however did not provide a satisfactory asthma control. Fifty-two patients were randomly treated with weekly subcutaneous administrations of dupilumab (300 mg) for 12 weeks, and 52 subjects were assigned to receive placebo. LABA therapy was interrupted after 4 weeks, whereas ICS treatment was progressively tapered and then suspended between weeks 6 and 9. The primary outcome of this study was to evaluate the impact of dupilumab on asthma exacerbations. Three patients in the dupilumab arm (6%) and 23 subjects in the placebo group (44%) experienced a disease exacerbation, respectively; this difference resulted to be statistically significant (*p* < 0.001), and dupilumab reduced the asthma exacerbation rate by 87%. In regard to the secondary endpoints, dupilumab enhanced forced expiratory volume in the first second (FEV_1_) by more than 200 ml, and also incremented the morning peak expiratory flow (PEF). Other improvements elicited by dupilumab included a decrease in the score of asthma control questionnaire (ACQ), as well as relevant reductions of nocturnal awakenings, evening and morning symptoms, and inhalation numbers of short-acting bronchodilators used as rescue medication. Furthermore, dupilumab significantly reduced airway and blood levels of several biomarkers of type 2 asthmatic inflammation, including fractional exhaled nitric oxide (FeNO), IgE, CCL26 (eotaxin-3), and TARC (thymus and activation-regulated chemokine). Conversely, dupilumab increased blood eosinophil numbers in 4 patients. With regard to safety and tolerability, in comparison to placebo dupilumab raised the frequency of injection-site reactions, headache, nasopharyngitis, and nausea. Only one patient referred the onset of a cutaneous rush, which promptly recovered after a mild treatment with systemic corticosteroids, and anti-histamines. Although this trial provided some valuable information, its study design was not adherent to a real-life setting (Wechsler, 2013; Vatrella et al., 2014). In fact, in daily clinical practice ICS/LABA combinations are not usually suspended during add-on biological treatment of asthma.

The above discrepancy was emended by the study protocol of a larger, subsequent double-blind, randomized, placebo-controlled, dose-ranging, and parallel-group phase 2b trial, performed in adult people with persistent asthma, uncontrolled by medium-to-high doses of ICS/LABA associations, which were not interrupted during add-on treatment with dupilumab (Wenzel et al., 2016). This study was articulated into three phases, consisting of a screening period of 14–21 days, followed by a randomized therapy course of 24 weeks, and by a subsequent post-treatment follow-up lasting 16 weeks. 776 patients were randomly subdivided into five groups, undergoing the following subcutaneous treatments: 1) placebo (158 subjects); 2) dupilumab, 200 mg every 2 weeks (150 subjects); 3) dupilumab, 200 mg every 4 weeks (154 subjects); 4) dupilumab, 300 mg every 2 weeks (157 subjects); 5) dupilumab, 300 mg every 4 weeks (157 subjects). Except for the study arm including patients treated with 200 mg of dupilumab every 4 weeks, in comparison to placebo all the other groups experienced significant FEV_1_ increments, which at the 24^th^ week were comprised between 0.15 and 0.16 L. Additionally, when injected at intervals of 2 weeks, dupilumab significantly decreased the annual numbers of severe asthma exacerbations. All drug dosages also elicited significant and dose-dependent reductions of FeNO, which were quantitatively more relevant when dupilumab was administered every 2 weeks. The effects of dupilumab on asthma exacerbations, pulmonary function and FeNO levels were independent of blood eosinophil counts. Dupilumab was characterized by a good safety and tolerability profile, as shown by the similar distribution across the five study groups of mild adverse events, mainly including reactions at injection site, upper respiratory tract infections, and headache. Temporary elevations of blood eosinophils were detected in some patients with baseline blood eosinophil numbers of at least 300 cells/μl.

The Liberty Asthma Quest trial was a phase 3 double-blind, randomized, placebo-controlled and parallel-group study, which evaluated the efficacy of dupilumab in uncontrolled moderate-to-severe asthma (Castro et al., 2018). In particular, 1902 patients aged at least 12 years were randomly partitioned into four groups, treated for 52 weeks with subcutaneous injections as follows: 1) first loading dosage (400 mg) of dupilumab, followed by a single dose of 200 mg every 2 weeks; 2) matched volume (1.14 ml) of placebo; 3) first loading dosage (600 mg) of dupilumab, followed by a single dose of 300 mg every 2 weeks; 4) matched volume (2 ml) of placebo. In comparison to placebo arms, patients included in both groups treated with dupilumab experienced a near 50% decrease in the annualized rate of severe asthma exacerbations. This reduction percentage overcame 65% in subjects with blood eosinophil counts of 300 or more cells/μl. Both dosages of dupilumab also induced significant FEV_1_ increases, which resulted to be even greater in patients with at least 300 blood eosinophils/μL. Moreover, dupilumab improved asthma symptom control, as shown by the significant decrements of asthma control questionnaire (ACQ)-5 scores. Furthermore, dupilumab significantly lowered several biomarkers of type 2 asthma, including serum IgE levels, FeNO, as well as the blood concentrations of eotaxin-3, periostin, and TARC. With regard to adverse events, transient increments of blood eosinophils were detected in 52 patients (4.1%) treated with dupilumab, and in 4 subjects (0.6%) who received placebo. A post-hoc analysis of this trial showed that dupilumab decreased severe asthma exacerbations and type 2 inflammatory biomarkers, and also improved asthma control and lung function regardless of the allergic status of treated patients (Corren et al., 2020).

The primary goal of the Liberty Asthma Venture study was to assess the corticosteroid-sparing effect of dupilumab (Rabe et al., 2018). In particular, this phase 3 double-blind, randomized, placebo-controlled trial enrolled 210 patients with oral corticosteroid-dependent severe asthma. These subjects were randomly assigned in a 1:1 ratio to receive for 24 weeks either placebo or an add-on biological therapy with dupilumab, consisting of a first loading dose of 600 mg, then followed by a subcutaneous injection of 300 mg every 2 weeks. Study results demonstrated that a 41.9% reduction of oral glucocorticoid dosage occurred in the placebo arm, whereas a 70.1% decrease was observed in the dupilumab group. In particular, when compared with the 25% rate of participants receiving placebo who suspended the oral intake of glucocorticoids, 48% of patients treated with dupilumab interrupted systemic corticosteroid therapy. In spite of either tapering or discontinuation of oral corticosteroid (OCS) use, in comparison to placebo dupilumab lowered by 59% the number of severe asthma exacerbations, and also enhanced FEV_1_ by 220 ml. A temporary blood eosinophilia was detected in 14% of patients treated with dupilumab, and in 1% of people belonging to the placebo group.

Many patients enrolled in the above trials were also recruited for an open-label extension study named TRAVERSE, which was carried out in 362 hospitals across 27 countries, thus monitoring the effects of dupilumab (first dosage of 600 mg, followed by a 300 mg dose administered every 2 weeks) for 96 weeks in 2,282 adults and adolescents with moderate-to-severe asthma (Wechsler et al., 2022). This study confirmed the clinical and functional therapeutic effects of dupilumab, mainly expressed by relevant decreases in asthma symptoms and exacerbations, paralleled by important FEV_1_ increases. However, the primary aim of TRAVERSE was to investigate the long-term safety of dupilumab. In this regard, dupilumab displayed a sustained profile characterized by good safety and tolerability patterns. The most common adverse events were nasopharyngitis, bronchitis, and erythema at injection site. The most frequent serious adverse events, occurring in very low percentages of patients, included asthma exacerbations and pneumonia. Anti-drug antibodies (ADAs) were detected in 157 patients, but did not have any impact on the efficacy and safety of dupilumab. With regard to the biomarkers of type-2 asthma, dupilumab progressively lowered the levels of both blood eosinophils, and serum total IgE.

The Liberty Asthma Voyage trial was a phase 3, double-blind, randomized, and placebo-controlled study which lasted 52 weeks and evaluated the therapeutic effects of dupilumab in children (age: 6–11 years) with moderate-to-severe asthma (Bacharier et al., 2021). Dupilumab was administered every 2 weeks as add-on biological therapy at doses of either 100 mg (body weight ≤30 kg) or 200 mg (body weight >30 kg). In particular, 408 asthmatic children were randomly assigned to receive dupilumab or placebo. When compared to placebo, dupilumab significantly reduced the annualized rate of severe asthma exacerbations and also bettered asthma symptom control, as shown by the improvement of the score of Asthma Control Questionnaire 7 Interviewer-Administered (ACQ-7-IA) at week 24. Moreover, add-on treatment with dupilumab had a positive impact on pulmonary function, as demonstrated by the significant increment at week 12 of the percentage of predicted pre-bronchodilator FEV_1_ (ppFEV_1_). With regard to the occurrence of serious adverse events, no difference was found between the dupilumab group and the placebo arm.

In addition to the above mentioned trials, which are summarized in Table 1, dupilumab has also been recently tested in real-life studies.

**TABLE 1:** Dupilumab in severe asthma: summary of the largest clinical trials.

Authors	No. Patients	Phase	Main results
Wenzel et al., 2013 (ref. 80)	104	2a	Fewer asthma exacerbations, higher FEV_1_
Wenzel et al., 2016 (ref. 82)	776	2b	Fewer asthma exacerbations, higher FEV_1_
Castro et al., 2018 (ref. 83)	1902	3	Fewer asthma exacerbations, better symptom control, higher FEV_1_
Rabe et al., 2018 (ref. 85)	210	3	Lower intake of oral corticosteroids, higher FEV_1_
Bacharier et al., 2021 (ref. 87)	408	3	Fewer asthma exacerbations, better symptom control, higher FEV_1_
Wechsler et al., 2022 (ref. 86)	2282	Open-label extension	Long-term safety, fewer asthma exacerbations, higher FEV_1_

A French multi-centre, nationwide, real-life retrospective study was conducted in 64 severe asthmatic patients, with the aim of verifying the effectiveness and safety of dupilumab (Dupin et al., 2020). After 12 months of treatment, the annual number of asthma exacerbations diminished from 4 to 1, and the score of the asthma control test (ACT) enhanced from 14 (poor symptom control) to 22 (good symptom control). Furthermore, the daily prednisone intake decreased from 20 to 5 mg, and the median value of FEV_1_ increased from 58 to 68% predicted, corresponding to a median increment of 200 ml. Skin reactions at injection-site were the most frequent side effects. Similar clinical and functional benefits were also observed during an investigational period of 1 year, referring to an Italian real-world multicentre study performed in severe asthmatic patients (Campisi et al., 2021).

In a real-life setting, we evaluated the rapidity of dupilumab efficacy in patients with severe asthma and nasal polyposis, who were examined at baseline and after only 4 weeks of treatment (Pelaia et al., 2021). Dupilumab quickly improved symptom control related to both severe asthma and nasal polyposis, as shown by the mean changes regarding the scores of ACT questionnaire (from 12 to 21) and sino-nasal outcome test-22 (SNOT-22: from 58 to 19), respectively. Such positive clinical achievements allowed to progressively taper, and then completely eliminate OCS consumption within 4 weeks. Moreover, in the same period we noticed that FEV_1_, peak expiratory flow (PEF), and forced mid-expiratory flow at 25–75% of forced vital capacity (FEF_25-75_) increased by more than 200 ml, 0.6 L/s, and 0.3 L/s, respectively. Such a relevant improvement of airflow limitation was associated with a significant reduction of lung hyperinflation, documented by parallel decreases of residual volume (RV: −690 ml) and total lung capacity (TLC: −460 ml). These last results are very important in consideration of the crucial contribution given by air trapping to the functional abnormalities characterizing severe asthma (Jarjour et al., 2012).

Dupilumab is also very effective as biological therapy of some relevant asthma comorbidities such as nasal polyposis and atopic dermatitis, characterized by type-2 inflammation (Ricciardolo et al., 2021). Indeed, the two phase 3 multicentre, double-blind, randomized, and placebo-controlled LIBERTY NP SINUS-24 and -52 trials showed that dupilumab significantly ameliorated nasal congestion and obstruction, as well as decreased nasal polyp size and paranasal sinus opacification (Bachert et al., 2019). Moreover, in asthmatic patients with comorbid atopic dermatitis, dupilumab can improve lung function, and reduce the eczema area (Benzecry et al., 2021).

## Concluding Remarks

The recent advances in phenotypic and endotypic characterization of severe asthma have paved the way for the development of excellent therapeutic tools within the context of biological treatments with monoclonal antibodies. In particular, the IL-4/IL-13 cytokine axis plays a pivotal pathogenic role in type-2 inflammation. Therefore, by effectively blocking at a receptor level the pro-inflammatory mechanisms driven by both IL-4 and IL-13, dupilumab provides valuable benefits for patients with severe type-2 asthma. Dupilumab is especially indicated in the presence of high levels of type-2 biomarkers such as eosinophils (blood cell count ≥150 cells/μl) and FeNO (≥25 ppb), eventually associated with OCS-dependence (Moran and Pavord, 2020). Furthermore, dupilumab seems to be very effective in both allergic and non-allergic severe asthma, and exerts its powerful pharmacologic action also when patients complain of asthma comorbidities such as atopic dermatitis and chronic rhinosinusitis with nasal polyps.
